# Confidence Analysis of Standard Deviational Ellipse and Its Extension into Higher Dimensional Euclidean Space

**DOI:** 10.1371/journal.pone.0118537

**Published:** 2015-03-13

**Authors:** Bin Wang, Wenzhong Shi, Zelang Miao

**Affiliations:** Department of Land Surveying and Geo-Informatics, The Hong Kong Polytechnic University, Kowloon, Hong Kong, China; Fondazione Edmund Mach, Research and Innovation Centre, ITALY

## Abstract

Standard deviational ellipse (SDE) has long served as a versatile GIS tool for delineating the geographic distribution of concerned features. This paper firstly summarizes two existing models of calculating SDE, and then proposes a novel approach to constructing the same SDE based on spectral decomposition of the sample covariance, by which the SDE concept is naturally generalized into higher dimensional Euclidean space, named standard deviational hyper-ellipsoid (SDHE). Then, rigorous recursion formulas are derived for calculating the confidence levels of scaled SDHE with arbitrary magnification ratios in any dimensional space. Besides, an inexact-newton method based iterative algorithm is also proposed for solving the corresponding magnification ratio of a scaled SDHE when the confidence probability and space dimensionality are pre-specified. These results provide an efficient manner to supersede the traditional table lookup of tabulated chi-square distribution. Finally, synthetic data is employed to generate the 1-3 multiple SDEs and SDHEs. And exploratory analysis by means of SDEs and SDHEs are also conducted for measuring the spread concentrations of Hong Kong’s H1N1 in 2009.

## Introduction

Standard deviation arises as one of the classical statistical measures for depicting the dispersion of univariate features around its center. Its evolution in two dimensional space arrives at the standard deviational ellipse (SDE), which was firstly proposed by Lefever [1] in 1926. Ever since then, SDE has long served as a versatile GIS tool for delineating the bivariate distributed features. It is typically employed for sketching the geographical distribution trend of the features concerned by summarizing both of their dispersion and orientation. Although SDE’s arrival once aroused great attention, a certain amount of consequent criticism followed as well, mainly due to the fact that Lefever’s defined curve is not an ellipse [2], but the standard deviation curve (SDC) as nominated by Gong [3].

Wide utilization potentialities exerted by SDE are extensively found in many research fields and commercial industries. For instance, Smith and Cheeseman [4] employ it for estimating the spatial uncertainty between coordinate frames representing the relative locations of a mobile robot. Besides, SDE has also been adopted to quantitatively analyze the orientation anisotropy in contaminant barrier particles [5], and explore the geographical distribution of household activity or travel behavior thereby promoting the policy formulation in response to urban travel reduction strategies [6]. Meanwhile, geographically profiling of the distributional trend for a series of crimes [7,8] by SDE might detect a relationship to particular physical features such as some restaurants or apartments and even the lairs of the criminals. Mapping groundwater well samples for some kind of contaminant could identify how and to what extent the toxin is spreading, which consequently, may be conducive to deploy the responding mitigation strategies [9]. Moreover, comparing the coverage area, shape, and overlap of ellipses for various racial or ethnic groups may provide insights regarding racial or ethnic segregation [10]. Furthermore, graphing ellipses for a disease outbreak such as malaria surveillance [11] over time can potentially make the real-time prediction of its spatial spread trend, since the central tendency and dispersion are two principal aspects attracting the concerns from epidemiologists.

As a GIS tool for delineating spatial point data, SDE is mainly determined by three measures: average location, dispersion (or concentration) and orientation. In addition to the traditional mean center (gravity of the distribution) suggested by Lefever [1], weighted mean or median could also be the alternative options, together with the weighted covariance of observations which evolve into some variants of the SDE [12]. It is worth noting that SDE also lays the foundation for many other advanced models, such as the minimum covariance determinant estimator (MCD) [13,14] for outlier detections and elliptic spatial scan statistic [15] employed in spatiotemporal disease surveillance. From the perspective of practical implementation, Alexandersson [16] once wrote an *ellip* command for graphing the confidence ellipses in Stata 8, though the latest version being Stata 13 already.

Although SDE has extensive applications in various fields ever since 1926, it still has not been correctly clarified sometimes. For instance, from the latest resources in ArcGIS Help 10.1 describing how standard deviational ellipse works, it is stated that one, two and three standard deviation(s) can encompass approximately 68%, 95% and 99% of all input feature centroids respectively, supposing the features concerned follow a spatially normal distribution. However, this content corresponds to the well-known 3-sigma rule with respect to univariate normal distribution, rather than bivariate case. Worse still, there is even an attached illustration therein depicting several bivariate geographical features located within a planar map. Obviously, such confusing interpretation may mislead the GIS users to believe the univariate 3-sigma rule remains valid in two-dimensional Euclidean space, or even higher dimensions.

For fully clarifying the implications of SDE, Sect. 2 below devotes to firstly summarizing two existing models of deriving the SDE’s calculation formulas, and secondly proposing a novel approach for constructing the same SDE based on spectral decomposition of the sample covariance, by which SDE concept is further extended into higher dimensional Euclidean space, named standard deviational hyper-ellipsoid (SDHE). Most of all, rigorous recursion formulas are then derived for calculating the confidence levels of scaled SDHE with arbitrary magnification ratios in any dimensional space. Besides, an inexact-newton method based iterative algorithm is also proposed for solving the corresponding magnification ratio of a scaled SDHE when the confidence probability and space dimensionality are pre-specified. Finally, synthetic data is employed to generate the 1–3 multiple SDEs and SDHEs in two and three dimensional spaces, respectively. Meanwhile, exploratory analysis by means of SDEs and SDHEs are also conducted for measuring the spread concentrations of Hong Kong’s H1N1 in 2009.

## Methods

First two subsections below devotes to a brief summarization of two classical approaches to generating the standard deviational ellipses in 2D. After that, a novel approach based on spectral decomposition of the covariance matrix is introduced which achieves the same calculation formula of SDE. This spectral decomposition based approach will be adopted for constructing the generalized standard deviational (hyper-)ellipsoids into higher dimensional Euclidean space in the next Sect. 3.

### 2.1 Explore the orientated data for extreme standard deviations

Standard deviational ellipse delineates the geographical distribution trend by summarizing both dispersion and orientation of the observed samples. There are already several approaches to obtaining the computational formula of SDE. The upcoming discussed method presented by Yuill [12] was actually a melioration of Lefever’s original model [1] despite of suffering from certain criticisms [2].

Suppose a series of independent identically distributed samples (***x***_i_, ***y***_i_), ***i*** = **1,**…***,n*** are drawn from a Gaussian population. A standard deviational ellipse can be determined according to the following steps. Firstly, make sample mean be the origin of new axes, thereby simultaneously centering all the observed samples,

$$\bar{x}=\frac{1}{n}\sum_{i=1}^{n}x_{i},\bar{y}=\frac{1}{n}\sum_{i=1}^{n}y_{i},\left(\begin{array}{c} {\tilde{x}}_{i}\\ {\tilde{y}}_{i} \end{array}\right)=\left(\begin{array}{c} x_{i}\\ y_{i} \end{array}\right)-\left(\begin{array}{c} \bar{x}\\ \bar{y} \end{array}\right).$$ (1)

**Fig 1:**
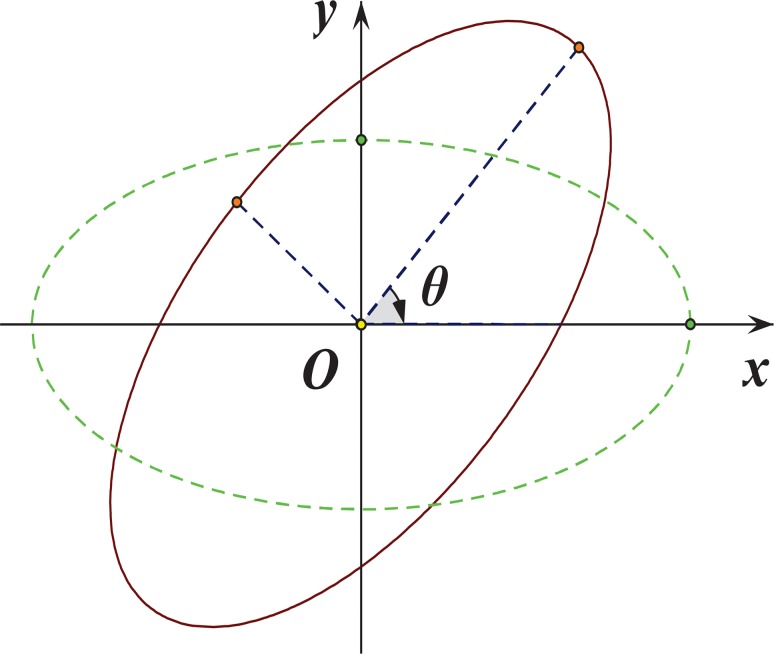
An ellipse rotated with an angle *θ* in clockwise direction.

Next, introduce a rotation matrix $G=\left(\begin{array}{cc} \cos\theta & \sin\theta \\ -\sin\theta & \cos\theta \end{array}\right)$ with an angle *θ* in clockwise direction as illustrated in Fig. 1, all observed sample points are then transformed into a new planar coordinate system,

$$\left(\begin{array}{c} {x^{\prime }}_{i}\\ {y^{\prime }}_{i} \end{array}\right)=G\left(\begin{array}{c} {\tilde{x}}_{i}\\ {\tilde{y}}_{i} \end{array}\right)=\left(\begin{array}{cc} \cos\theta & \sin\theta \\ -\sin\theta & \cos\theta \end{array}\right)\left(\begin{array}{c} {\tilde{x}}_{i}\\ {\tilde{y}}_{i} \end{array}\right)=(\begin{array}{c} {\tilde{y}}_{i}\sin\theta +{\tilde{x}}_{i}\cos\theta \\ {\tilde{y}}_{i}\cos\theta -{\tilde{x}}_{i}\sin\theta \end{array}).$$ (2)

The maximum likelihood estimator [17] of the rotated samples’ variance yields,

$$\left\{ \begin{array}{l} \sigma_{x^{\prime }}^{2}=\frac{1}{n}\sum_{i=1}^{n}{\left({x^{\prime }}_{i}\right)}^{2}=\frac{1}{n}\sum_{i=1}^{n}{\left({\tilde{y}}_{i}\sin\theta +{\tilde{x}}_{i}\cos\theta \right)}^{2}\\ \sigma_{y^{\prime }}^{2}=\frac{1}{n}\sum_{i=1}^{n}{\left({y^{\prime }}_{i}\right)}^{2}=\frac{1}{n}\sum_{i=1}^{n}{\left({\tilde{y}}_{i}\cos\theta -{\tilde{x}}_{i}\sin\theta \right)}^{2} \end{array}.\right.$$ (3)

Consequently, corresponding angles for producing the maximum and minimum standard deviations can be obtained by equating any derivative of the above variance estimators w.r.t. *θ* to be zero [5,12], that is

$$\frac{d\sigma_{x^{\prime }}^{2}}{d\theta }=\frac{2}{n}\sum_{i=1}^{n}\left({\tilde{y}}_{i}^{2}\sin\theta \cos\theta +{\tilde{x}}_{i}{\tilde{y}}_{i}\left(\cos^{2}\theta -\sin^{2}\theta \right)-{\tilde{x}}_{i}^{2}\sin\theta \cos\theta \right)=0.$$

According to Vieta's formulas, general solution to the above quadratic equation is

$$\tan\theta =\frac{\left(\sum_{i=1}^{n}{\tilde{x}}_{i}^{2}-\sum_{i=1}^{n}{\tilde{y}}_{i}^{2}\right)\pm \sqrt{{\left(\sum_{i=1}^{n}{\tilde{x}}_{i}^{2}-\sum_{i=1}^{n}{\tilde{y}}_{i}^{2}\right)}^{2}+4{\left(\sum_{i=1}^{n}{\tilde{x}}_{i}{\tilde{y}}_{i}\right)}^{2}}}{2\sum_{i=1}^{n}{\tilde{x}}_{i}{\tilde{y}}_{i}}.$$ (4)

Each of these two angles corresponds to the maximum and minimum deviation in the new coordinate system, respectively. By merging Eq. (4) into Eq. (3), the major axis and minor axis of SDE can be determined for measuring the dispersion distribution of original observations.

It should be noticed that rotating $\sigma_{x^{\prime }}^{2}$ in Eq. (3) around the sample mean center defines an implicit locus curve [1]. However, such a closed curve is not an ellipse [2], but actually the standard deviation curve (SDC) nominated by Gong [3] with its expression as follows,

$${\left({\tilde{x}}^{2}+{\tilde{y}}^{2}\right)}^{2}=\sigma_{x}^{2}{\tilde{x}}^{2}+2\rho \sigma_{x}\sigma_{y}\tilde{x}\tilde{y}+\sigma_{y}^{2}{\tilde{y}}^{2}.$$ (5)

**Fig 2:**
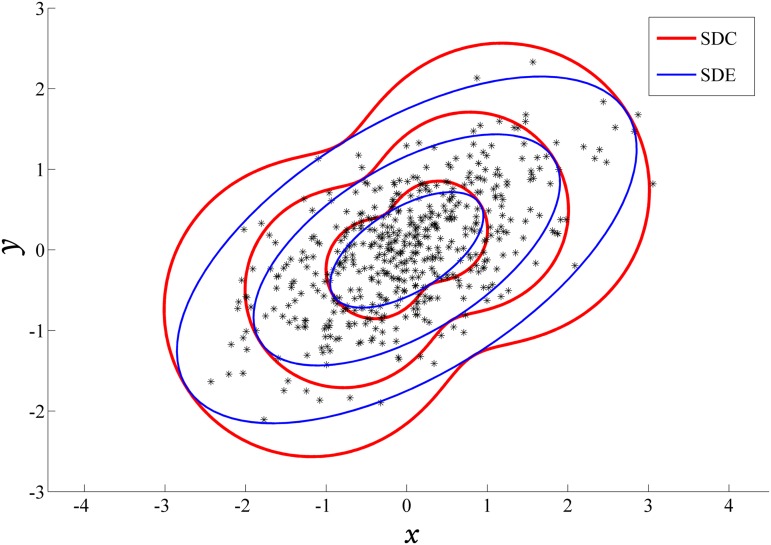
One synthetic experiment of SDC and SDE constructed upon 500 sampling points from a bivariate normal distribution.

Here ***ρ*** is the correlation coefficient between ***x*** and ***y*** coordinates. For seeking a striking contrast between SDC and SDE, a numerical experiment is conducted, employing 500 synthetic points extracted from a bivariate normal variable with mean ***μ*** = **(0,0)** ^T^and covariance matrix$C=\left(\begin{array}{cc} 0.9 & 0.4\\ 0.4 & 0.5 \end{array}\right)$. Based on these sampling points, contradistinctive profiles of 1–3 multiple SDC and SDE are illustrated in Fig. 2. Conspicuously there are 4 tangency points for each corresponding pair, and SDC appears occupying an overall larger area then SDE.

### 2.2 Optimal linear central tendency measure

Another method described by Cromley [18] aims to explore such an optimal linear central tendency measure, ***ax*+*by*+*c* = 0**, which passes through the distributed samples. This is equivalent to an optimization problem with objective of minimizing the summation of total perpendicular distances from any observation point to this line subject to the constraint of ***a*** ^**2**^ **+*b*** ^**2**^ **= 1**, which guarantees the scale invariance, namely,

$$\begin{array}{ll} \min & \sum_{i=1}^{n}{\left(ax_{i}+by_{i}+c\right)}^{2}\\ s.\;t. & a^{2}+b^{2}=1 \end{array}.$$ (6)

The above constrained optimization problem can be solved by Lagrangian multiplier method, yielding the optimal linear central tendency which precisely coincides with the direction of principal axis of SDE. Therefore, solution to the above optimization arrives at exactly the same calculation formulas of SDE as the aforementioned first approach.

### 2.3 Spectral decomposition of the covariance matrix

Using the symbols introduced in Eq. (1), this subsection devotes to present another approach for constructing SDE by means of spectral decomposition of the sample covariance matrix, which is formulated as

$$C=\left(\begin{array}{cc} \mathrm{var}\left(x\right) & \text{cov}\left(x,y\right)\\ \text{cov}\left(y,x\right) & \mathrm{var}\left(y\right) \end{array}\right)=\frac{1}{n}\left(\begin{array}{cc} \sum_{i=1}^{n}{\tilde{x}}_{i}^{2} & \sum_{i=1}^{n}{\tilde{x}}_{i}{\tilde{y}}_{i}\\ \sum_{i=1}^{n}{\tilde{x}}_{i}{\tilde{y}}_{i} & \sum_{i=1}^{n}{\tilde{y}}_{i}^{2} \end{array}\right),$$ (7)

where $\mathrm{var}\left(x\right)=\frac{1}{n}\sum_{i=1}^{n}{\left(x_{i}-\bar{x}\right)}^{2}=\frac{1}{n}\sum_{i=1}^{n}{\tilde{x}}_{i}^{2}$,$cov\left(x,y\right)=\frac{1}{n}\sum_{i=1}^{n}\left(x_{i}-\bar{x}\right)\left(y_{i}-\bar{y}\right)=\frac{1}{n}\sum_{i=1}^{n}{\tilde{x}}_{i}{\tilde{y}}_{i}$ and$\mathrm{var}\left(y\right)=\frac{1}{n}\sum_{i=1}^{n}{\left(y_{i}-\bar{y}\right)}^{2}=\frac{1}{n}\sum_{i=1}^{n}{\tilde{y}}_{i}^{2}$.

It must be said there are two common textbook definitions of variance and covariance, as well as the standard deviation. One is the unbiased estimator while the other one is the maximum likelihood estimator proved by Li and Racine [17]. Their calculation formulas differ only in ***n***-**1** versus ***n*** in the divisor. To keep consistent with the previous equations involved, the latter estimator is employed hereafter.

After spectral decomposition of the sample covariance (7), SDE can be constructed by assigning square roots of eigenvalues as the lengths of its semi-major and semi-minor axes [19], to which being parallel by the corresponding eigenvectors. Solving of the characteristic polynomial equation of covariance matrix ***C***, namely,

$$f\left(\lambda \right)=\det\left(\lambda I-C\right)=\det\left(\begin{array}{cc} \lambda -\frac{1}{n}\sum_{i=1}^{n}{\tilde{x}}_{i}^{2} & -\frac{1}{n}\sum_{i=1}^{n}{\tilde{x}}_{i}{\tilde{y}}_{i}\\ -\frac{1}{n}\sum_{i=1}^{n}{\tilde{x}}_{i}{\tilde{y}}_{i} & \lambda -\frac{1}{n}\sum_{i=1}^{n}{\tilde{y}}_{i}^{2} \end{array}\right)=0,$$ (8)

yields the lengths of the SDE’s semi-major and semi-minor axes, which are

σ1,2=((∑i=1nx˜i2+∑i=1ny˜i2)±(∑i=1nx˜i2−∑i=1ny˜i2)2+4(∑i=1nx˜iy˜i)22n)12, (9)

Meanwhile, one group of base vectors from the characteristic vector space satisfying Eq. (8) can be obtained by

$$v_{1,2}={\left(\left(\sum_{i=1}^{n}{\tilde{x}}_{i}^{2}-\sum_{i=1}^{n}{\tilde{y}}_{i}^{2}\right)\pm \sqrt{{\left(\sum_{i=1}^{n}{\tilde{x}}_{i}^{2}-\sum_{i=1}^{n}{\tilde{y}}_{i}^{2}\right)}^{2}+4{\left(\sum_{i=1}^{n}{\tilde{x}}_{i}{\tilde{y}}_{i}\right)}^{2}},\;2\sum_{i=1}^{n}{\tilde{x}}_{i}{\tilde{y}}_{i}\right)}^{\text{T}}.$$ (10)

Thus, it takes no effort to verify that orientation angles intersected by the principle axes of SDE and the planar coordinate axes are exactly the same, namely, the optimal angle appeared in Eq. (4).

In conclusion, the above three approaches actually all calculate the same SDE according to formulas (1), (4) and (9), respectively, which lays the theoretical basis for SDE to be one functional component in the Spatial Statistics toolbox of ArcGIS 10.1.

## Results

In Sect. 2, three approaches for constructing SDE have been summarized and compared upon the distributed samples in two-dimensional space. This section will generalize the SDE concept into higher dimensional Euclidean space, yielding the standard deviational hyper-ellipsoid (SDHE), be means of the spectral decomposition of covariance matrix. Meanwhile, rigorous mathematical derivations attempt to figure out the relationship between the confidence levels characterizing the probabilities of random scattered points falling inside a scaled SDHE and the corresponding magnification ratio under the assumption that samples follow Gaussian distribution.

### 3.1 Construction of Standard Deviational Hyper-Ellipsoid

Suppose ***S***∈***R*** ^n^ be an n-dimensional Gaussian random vector, that is ***S***~***N*** *(* ***μ*,*C***) with its probability density function

f(s)=1(2π)n2|C|12exp{−12(s−μ)TC−1(s−μ)}. (11)

And ***S***_1_,***S***_2_,…, ***S***_m_ represent ***m*** independent and identically distributed samples extracted from population ***S***. In general, the maximum likelihood estimators [17] for parameters ***μ*** and ***C*** employed in Eq. (11) can be given by

$$\hat{\mu }=\frac{1}{m}\sum_{i=1}^{m}S_{i},\hat{C}=\frac{1}{m}\sum_{i=1}^{m}\left(S_{i}-\hat{\mu }\right){\left(S_{i}-\hat{\mu }\right)}^{\text{T}}.$$ (12)

Since covariance matrix ***C*** is real symmetric (positive semi-definite), there exists an orthogonal matrix ***Ԛ*** (formed by eigenvectors of ***C***) complying with the spectral decomposition,

$$C=QDQ^{\text{T}}.$$ (13)

Without loss of generality, suppose al the main diagonal elements of ***D*** = **diag(σ**_***i***_ **), *i*** = **1,2,**…,***n*** have been sorted in descending order, **σ**_**1**_ **≥σ**_**2**_≥…≥**σ**_**n**_. Due to the symmetry of covariance matrix ***C***, its spectral decomposition is actually equivalent to its singular value decomposition which output a series of automatically sorted eigenvalues (singular values). As thus, mapping a unit sphere by square root of covariance matrix, C12, yields a standard hyper-ellipsoid, with eigenvalues to be its principle semi-axes oriented by their corresponding eigenvectors [20].

Proceeding in this way, now comes to such an interesting question: how could this SDHE defined by Eq. (13) be represented graphically? This can be figured out by means of the Mahalanobis transformation [19] which is defined as

T=C−12(S−μ)=QD−12QT(S−μ). (14)

It can be verified that ***T***~***N***(0,***I***_***n***_) In other words, Mahalanobis transformation eliminates correlation between the variables and standardizes each variable with variance. Apparently, random vector ***T***’s SDHE happens to be a unit sphere (${\left\|T\right\|}_{2}=1$) in view of its isotropic distribution along any direction. Therefore, SDHE of original random vector ***S*** can be constructed from the transformation of a unit sphere by firstly stretching with a ratio of $\sqrt{\sigma_{i}}$ along each axis successively, then rotating the ellipsoid by orthogonal matrix ***Ԛ*** and a final translation of distribution center ***μ*** according to the following inverse Mahalanobis transformation,

S=QD12QTT+μ. (15)

### 3.2 Confidence level analysis of SDHE

This section settles the relationship between confidence levels characterizing the probabilities of random scattered points falling inside the scaled ellipsoids and the corresponding magnification ratio of such an SDHE by means of the rigorous mathematical formulas derivations.

The following scalar quantity

$$r^{2}={\left(S-\mu \right)}^{\text{T}}C^{-1}(S-\mu ),$$ (16)

is known as the Mahalanobis distance of the vector ***S*** away from its mean ***μ***. By merging Eqs. (13) and (14) into Eq. (16), it can be easily perceived that the above defined quadratic function is exactly the magnified SDHE with a magnification ratio of ***r*** and follows the chi-square distribution with ***n*** degrees of freedom,

$$\mathrm{Pr}\left\{r^{2}\leq \chi_{n,p}^{2}\right\}=p.$$ (17)

Table lookup of a tabulated chi-square distribution is always adopted as the traditional approach to acquire the exact confidence levels. Therefore, exploring to what extent the scattered samples obeying a Gaussian distribution is equivalent to examining whether they are falling inside such a scaled ellipsoid defined in terms of Eq. (16). Actually, calculation of the cumulative distribution function of chi-square distribution for a prescribed value ***x*** and the degrees-of-freedom ***n***, namely, $F\left(x|n\right)=\int_{0}^{x}\frac{t^{\frac{n}{2}-1}e^{-\frac{t}{2}}}{2^{\frac{n}{2}}\Gamma \left(\frac{n}{2}\right)}dt$, is eventually transformed to calculate the gamma density function with parameters n/2 and 2 in computer implementation, since chi-square distribution can be perceived as one child of the gamma distribution family with two varying parameters. Knüsel [21] has proposed a numerical algorithm with some supplement functions and a specified relative accuracy, which has been adopted in many modern statistical softwares, such as Matlab and R language. However, even using this algorithm, computation of the gamma density function is still extremely complex.

As mentioned above, SDE serves as a versatile spatial statistical tool for measuring the geographical distribution of features. Because of this, it has been embedded into many commercial software, like ArcGIS and Stata [16]. As a result, the algorithm’s practicability including the simplicity, speed and precision are of particular concern, which also originally stimulates us pursuing for an innovative approaches. In the subsequent portion, recursion formulas are derived for calculating the confidence levels and an iterative algorithm is proposed for solving the corresponding magnification ratio of the scaled ellipsoids after the prescribed scaling ratio or confidence level is given.

**3.2.1 The confidence level defined by a scaled SDHE.** Here an innovative recursion formula is presented by means of the multiple integral method for calculating the confidence level ***P***_***n***_ **(*r*)** of a scaled SDHE specified with a magnification factor ***r*** in ***n*** dimensional space so as to estimate the distribution of a random vector ***S***~***N***(***μ***,***C***), which is equivalent to the confidence level value of ***T***~***N***(**0**,***I***_***n***_), whose confidence region is exactly a sphere as explained in subsection 3.1; namely,

$$\mathrm{Pr}\left\{{\left(S-\mu \right)}^{\text{T}}C^{-1}\left(S-\mu \right)\leq r^{2}\right\}=\mathrm{Pr}\{T^{\text{T}}T\leq r^{2}\}.$$

**Fig 3:**
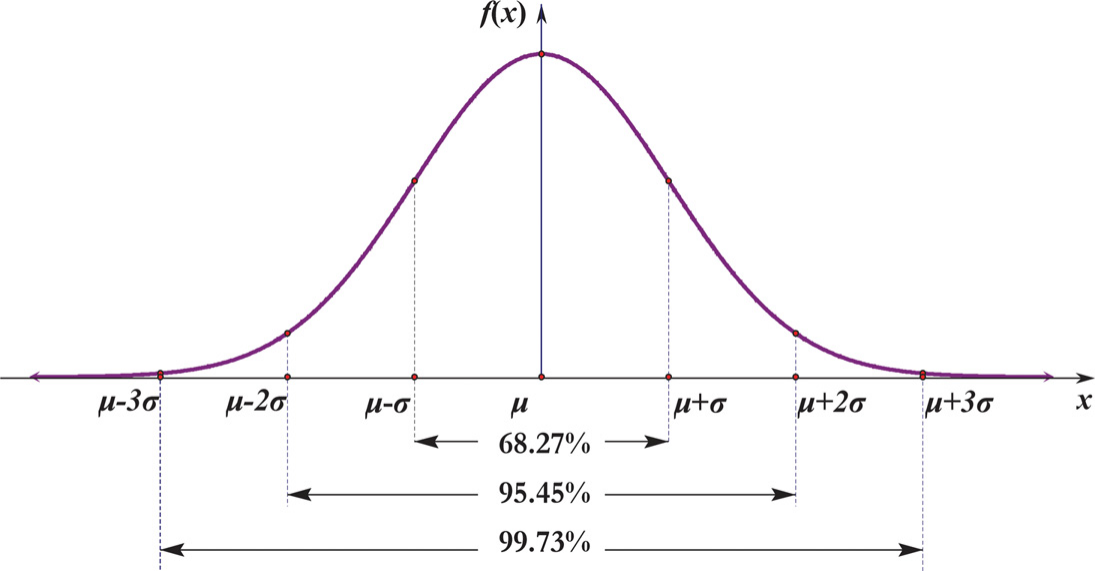
The confidence intervals correspond to 3-sigma rule of the normal distribution.

Therefore, for 1D case,

$$\begin{array}{l} P_{1}\left(r\right)=\mathrm{Pr}\left\{X_{1}{}^{\text{T}}X_{1}\leq r^{2}\right\}=\int_{-r}^{r}\frac{1}{\sqrt{2\pi }}e^{-\frac{x^{2}}{2}}ds\\ =\frac{2}{\sqrt{\pi }}\int_{0}^{r}e^{-\frac{x^{2}}{2}}d\left(\frac{x}{\sqrt{2}}\right)=\frac{2}{\sqrt{\pi }}\int_{0}^{\frac{r}{\sqrt{2}}}e^{-t^{2}}dt=\text{erf}\left(\frac{r}{\sqrt{2}}\right) \end{array};$$ (18)

where the error function is defined as $\text{erf}\left(x\right)=\frac{2}{\sqrt{\pi }}\int_{0}^{x}e^{-t^{2}}dt$, with another name being Gauss error function [22], which is a non-elementary function of sigmoid shape constantly occurring in probability, statistics and partial differential equations. As a matter of fact, Eq. (18) formulates the well-known 3-sigma rule of the most common normal distribution as illustrated in Fig. 3.

For 2D case,

$$\begin{array}{l} P_{2}\left(r\right)=\mathrm{Pr}\left\{X_{2}{}^{\text{T}}X_{2}\leq r^{2}\right\}=\iint_{x_{1}^{2}+x_{2}^{2}\leq r^{2}}{\left(\frac{1}{\sqrt{2\pi }}\right)}^{2}e^{-\frac{x_{1}^{2}+x_{2}^{2}}{2}}dx_{1}dx_{2}\\ =\frac{1}{2\pi }\int_{0}^{2\pi }\int_{0}^{r}re^{-\frac{r^{2}}{2}}drd\theta =1-e^{-\frac{r^{2}}{2}} \end{array};$$ (19)

**Fig 4:**
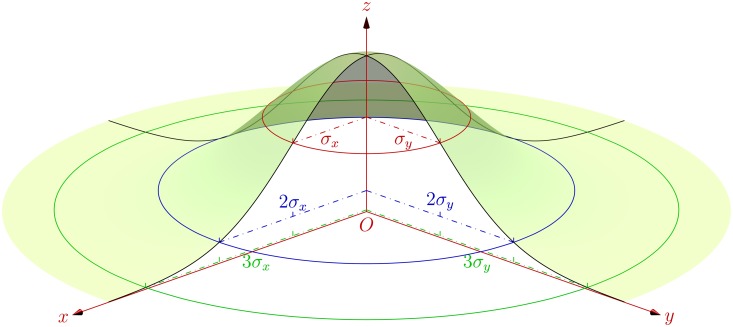
The confidence regions corresponds to 1–3 multiples of SDEs.

Hereinto, the polar coordinate transformation is introduced for above the penultimate equal sign. Next, the following Fig. 4 demonstrates the confidence ellipses corresponding to 1–3 multiples of SDEs in the color of red, blue and green, respectively.

It’s worth noting that an inverse formula here exists,

$$r=\sqrt{-2\ln\left(1-p\right)}.$$ (20)

for determining the magnification factor ***r*** which corresponds to a prescribed confidence level.

Before proceeding to the general formulas applicable in ***n*** dimensional space, we introduce the cubature formula [23] firstly, which calculates the volume of the ***n***-sphere of radius ***r***, with the quantity proportional to its ***n*** th power as follows,

$$V_{n}\left(r\right)=\frac{\pi^{\frac{n}{2}}}{\Gamma \left(\frac{n}{2}+1\right)}r^{n}.$$ (21)

Accordingly, for a general dimensional number ***n*≥3**,

$$\begin{array}{l} P_{n}\left(r\right)=P\left\{X_{n}{}^{\text{T}}X_{n}\leq r^{2}\right\}=\underset{\sum_{i=1}^{n}x_{i}^{2}\leq r^{2}}{\iint \cdots \int }{\left(\frac{1}{\sqrt{2\pi }}\right)}^{n}e^{-\frac{\sum_{i=1}^{n}x_{i}^{2}}{2}}dx_{1}dx_{2}\cdots dx_{n}\\ =\underset{\sum_{i=3}^{n}x_{i}^{2}\leq r^{2}}{\iint \cdots \int }{\left(\frac{1}{\sqrt{2\pi }}\right)}^{n-2}e^{-\frac{\sum_{i=3}^{n}x_{i}^{2}}{2}}\left(\iint_{x_{1}^{2}+x_{2}^{2}\leq r^{2}-\sum_{i=3}^{n}x_{i}^{2}}{\left(\frac{1}{\sqrt{2\pi }}\right)}^{2}e^{-\frac{x_{1}^{2}+x_{2}^{2}}{2}}dx_{1}dx_{2}\right)dx_{3}\cdots dx_{n}\\ ≐\underset{\sum_{i=3}^{n}x_{i}^{2}\leq r^{2}}{\iint \cdots \int }{\left(\frac{1}{\sqrt{2\pi }}\right)}^{n-2}e^{-\frac{\sum_{i=3}^{n}x_{i}^{2}}{2}}\left(1-e^{-\frac{r^{2}-\sum_{i=3}^{n}x_{i}^{2}}{2}}\right)dx_{3}\cdots dx_{n}\\ =\underset{\sum_{i=3}^{n}x_{i}^{2}\leq r^{2}}{\iint \cdots \int }{\left(\frac{1}{\sqrt{2\pi }}\right)}^{n-2}e^{-\frac{\sum_{i=3}^{n}x_{i}^{2}}{2}}ds_{3}\cdots ds_{n}-\underset{\sum_{i=3}^{n}x_{i}^{2}\leq r^{2}}{\iint \cdots \int }{\left(\frac{1}{\sqrt{2\pi }}\right)}^{n-2}e^{-\frac{r^{2}}{2}}dx_{3}\cdots dx_{n}\\ ≐P_{n-2}-{\left(\frac{1}{\sqrt{2\pi }}\right)}^{n-2}e^{-\frac{r^{2}}{2}}\cdot V_{n-2}\left(r\right)=P_{n-2}-{\left(\frac{1}{\sqrt{2\pi }}\right)}^{n-2}e^{-\frac{r^{2}}{2}}\cdot \frac{\pi^{\frac{n-2}{2}}}{\Gamma \left(\frac{n}{2}\right)}r^{n-2}\\ =P_{n-2}\left(r\right)-{\left(\frac{r}{\sqrt{2}}\right)}^{n-2}\frac{e^{-\frac{r^{2}}{2}}}{\Gamma \left(\frac{n}{2}\right)} \end{array}.$$ (22)

Hereinto, Γ is the gamma function, with some useful properties: $\Gamma \left(\frac{1}{2}\right)=\sqrt{\pi }$, Γ(1) = 1 and Γ **(*x*+1) = (*x*)**Γ**(*x*)** It should be noted that the first ≐ comes according to the results for 2D case in terms of Eq. (19) and the second ≐ follows Eq. (21) representing a sphere’s volume with radius ***r*** and dimensionality of ***n***-**2** Therefore, Eq. (22) totally characterizes the confidence probability for an arbitrary magnified SDHE with any specified magnification factor ***r*** in the form of a recursive formula applicable in any Euclidean space with dimensionality greater than 2. Similar findings regarding the confidence ellipse in terms of dimensionality ***n*** less than 3 have been provided in the appendix section of Smith and Cheeseman’s article [4]. However, to our knowledge, there is no precedent of such analytical expression of confidence levels for an ellipsoid in higher dimensional Euclidean space.

Computation of confidence levels using Eq. (22) is rather simple and efficient. There is only some algebraic manipulations and calculation of the supplement error function **erf (*x*)** if ***n*** is assigned to be an odd number. For better quantitatively perceiving the confidence levels of these scaled ellipsoids, the following Table 1 lists probability values corresponding to the scaled SDHEs which are magnified with different integer multiples from 1 to 7 and the space dimensionality not exceeding 10.

**Table 1:** Confidence levels of scaled SDHE vary with different magnification factors in spaces with the dimensionality not exceeding 10.

Dimensionality	Magnification factor						
	1	2	3	4	5	6	7
1	0.6827	0.9545	0.9973	0.9999	1.0000	1.0000	1.0000
2	0.3935	0.8647	0.9889	0.9997	1.0000	1.0000	1.0000
3	0.1987	0.7385	0.9707	0.9989	1.0000	1.0000	1.0000
4	0.0902	0.5940	0.9389	0.9970	0.9999	1.0000	1.0000
5	0.0374	0.4506	0.8909	0.9932	0.9999	1.0000	1.0000
6	0.0144	0.3233	0.8264	0.9862	0.9997	1.0000	1.0000
7	0.0052	0.2202	0.7473	0.9749	0.9992	1.0000	1.0000
8	0.0018	0.1429	0.6577	0.9576	0.9984	1.0000	1.0000
9	0.0006	0.0886	0.5627	0.9331	0.9970	1.0000	1.0000
10	0.0002	0.0527	0.4679	0.9004	0.9947	0.9999	1.0000

Observed from Table 1, 1-3 SDE(s) can encompass approximately 39.35%, 86.47% and 98.89% of all input feature centroids assuming these features follow a planar Gaussian distribution. It is evidently different from the content of our familiar 3-sigma rule. This finding can be conducive to clarify the confusing interpretation of confidence level regarding directional distribution in ArcGIS Help 10.1.

**3.2.2 The corresponding magnification factor to a prescribed confidence level.** Conversely, what size of a magnified SDHE can encompass the scattered features with a prescribed confidence probability? In other words, How to find the magnification factor ***r*** corresponding to a specified confidence level ***p*** in ***n*** dimensional space? This question can be answered by solving the following equation,

$$F\left(r\right)=P_{n}\left(r\right)-p,$$ (23)

with its derivative to be

$$F^{\prime }\left(r\right)={P^{\prime }}_{n}\left(r\right)=\{\begin{array}{ll} \sqrt{\frac{2}{\pi }}e^{-\frac{r^{2}}{2}} & n=1\\ re^{-\frac{r^{2}}{2}} & n=2\\ {P^{\prime }}_{n-2}\left(r\right)+\frac{r^{n-3}e^{-\frac{r^{2}}{2}}}{2^{\frac{n}{2}-1}\Gamma \left(\frac{n}{2}\right)}\left(r^{2}-n+2\right) & n\geq 3 \end{array}.$$ (24)

Thus, the approximate scaling ratio ***r*** can be solved according to the following iterative algorithm, which is put forward based on Newton method with Armijo rule [24].

**Algorithm 1 nsolg(** *r*_0_,*n*_0_,*p*,*τ*_*a*_,*τ*_*r*_ **)**

Evaluate *F(r*_*0*_ *) = P*_*n*_(r_0_)-*p*; *τ←τ*_*a*_ *+τ*_*r*_ *|F(r)|*

**While** *|F*(*r*)*|>τ* **Do**

Calculate the Newton direction *d = -F*'(*r*)^-1^ *F*(*r*) using (23)~(24), set *λ* = 1.

**While** *|F*(*r+λd*)*|>*(1-α*λ*)*|F*(*r*)*|* **Do**

*λ←σλ* where $\sigma \in \left[\frac{1}{10},\frac{1}{2}\right]$ is the reduction factor of the line search computed by minimizing a quadratic polynomial model *φ*(*λ*) **=** *|F*(*r+λd*)*|* ^*2*^

End While

r←r+λd

End While

Input arguments for this algorithm are the initial iterate ***r***_0_ with default value $\sqrt{n-1}$ which is an approximation of inflection point of the S-shape cumulative density function, space dimensionality ***n***, confidence level ***p***, relative and absolute termination tolerances $\tau_{a}=\tau_{r}=\sqrt{\varepsilon_{\text{machine}}}$ which need to be prescribed beforehand. Approximate solution with high accuracy can be soon obtained after a few iterations using this algorithm. Table 2 has tabulated the magnification ratios of scaled SDHEs for some commonly used confidence levels with space dimensionality not exceeding 10.

**Table 2:** Magnification ratios of scaled SDHE corresponding to different specified confidence levels with space dimensionality not exceeding 10.

Dimensionality	Confidence Level (%)					
	80.0	85.0	90.0	95.0	99.0	99.9
1	1.2816	1.4395	1.6449	1.9600	2.5758	3.2905
2	1.7941	1.9479	2.1460	2.4477	3.0349	3.7169
3	2.1544	2.3059	2.5003	2.7955	3.3682	4.0331
4	2.4472	2.5971	2.7892	3.0802	3.6437	4.2973
5	2.6999	2.8487	3.0391	3.3272	3.8841	4.5293
6	2.9254	3.0735	3.2626	3.5485	4.1002	4.7390
7	3.1310	3.2784	3.4666	3.7506	4.2983	4.9317
8	3.3212	3.4680	3.6553	3.9379	4.4822	5.1112
9	3.4989	3.6453	3.8319	4.1133	4.6547	5.2799
10	3.6663	3.8123	3.9984	4.2787	4.8176	5.4395

Seen from Table 2, the corresponding magnification factors become larger and larger along with the increase of space dimensionality, indicating that only bigger magnified ellipsoids can maintain the same prescribed confidence level in higher dimensional space compared with the counterpart in lower dimensional space.

## Experiments

### 4.1 Synthetic data experiments

In this section, two groups of synthetic data are employed to generate the 1–3 multiple SDEs and SDHEs in two and three dimensional spaces, respectively, to depict their aggregation extent and demonstrate the relationship between the scaled ellipse (or ellipsoid) size and their corresponding confidence levels.

**4.1.1 2D case.** Suppose that a series of scattered points ***X***_**i**_ ϵ *R* ^2^ are randomly generated from a two dimensional Gaussian vector, that is *X*_i_~***N***(***μ,C***). The following example employs 100 points with mean ***μ*** = **(2,3)** ^T^, and covariance $C=\left(\begin{array}{cc} 0.9 & 0.2\\ 0.2 & 0.5 \end{array}\right)$. Overlaying upon these scattered samples, 1–3 multiple SDEs are then created in terms of Eqs. (7)~(10) encompassing their geographic distribution with corresponding confidence degrees listed in Table 1.

For a better visualization of SDEs in computer imaging, the observed samples can be overlaid by a warning coloration, for example a (gradually varied) red layer processed with a transparency function. Intuitionally it should be inversely proportional to the confidence probability density of the features. By incorporating Eq. (16) into (11), an desirable transparency function can be of the following form,

$$f=1-e^{-\frac{r^{2}}{2}}.$$ (25)

**Fig 5:**
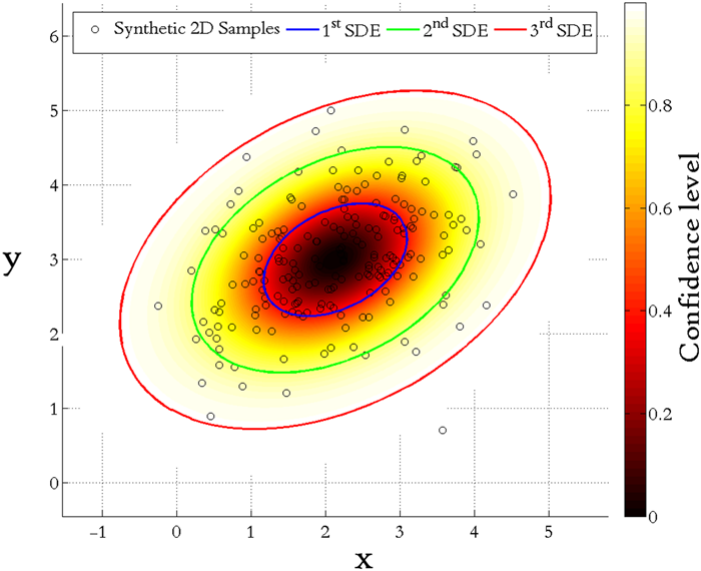
Visualization of 1–3 multiple SDEs for 2D scattered points.

This function can also be considered as a projection of the Gaussian probability density function upon the sample space. In the end, Fig. 5 presents a visualization of 1–3 multiple SDEs for these 2D scattered points.

**Fig 6:**
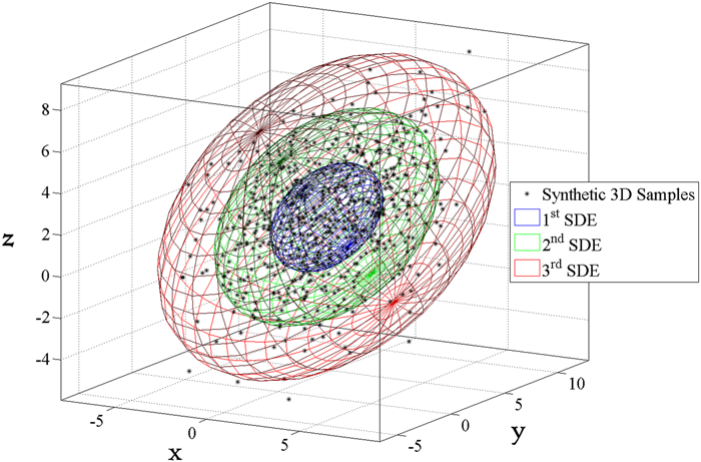
Visualization of 1–3 multiple SDEs for 3D scattered points.

**4.1.2 3D case.** Once again, suppose that a series of scattered points ***X***_***i***_ϵ*R* ^3^ are randomly generated, following 3D Gaussian distribution, that is *X*_i_ ~ ***N***(***μ,C***) The following example employs 600 points with mean ***μ*** = **(1,2,3)** ^T^, and covariance $C=\left(\begin{array}{ccc} 8 & -2 & 1\\ -2 & 8 & 2\\ 1 & 2 & 5 \end{array}\right)$. Based on these data samples, Fig. 6 exhibits 1–3 multiple SDEs constructed in terms of Eqs. (12)~(15) encompassing their geographic distribution with corresponding confidence degrees as listed in Table 1.

### 4.2 Spread analysis in Hong Kong’s H1N1 infections

The spread of epidemic diseases causes both very serious life risks and social-economic risks. For example, the latest epidemic outbreak in Hong Kong was Swine Flu Virus A (H1N1) causing hundreds of deaths and making all the residents get into a panic of fatal infection.

Geographic information science (GIS) serves as a common platform for convergence of disease surveillance activities. As one of its significant functional components, SDE, as well as SDHE, can be served to understand how the disease distributes together with its evolutionary trend, thereby assisting the epidemiologists or public health officials raising more effective strategies so as to control the disease spread.

**Fig 7:**
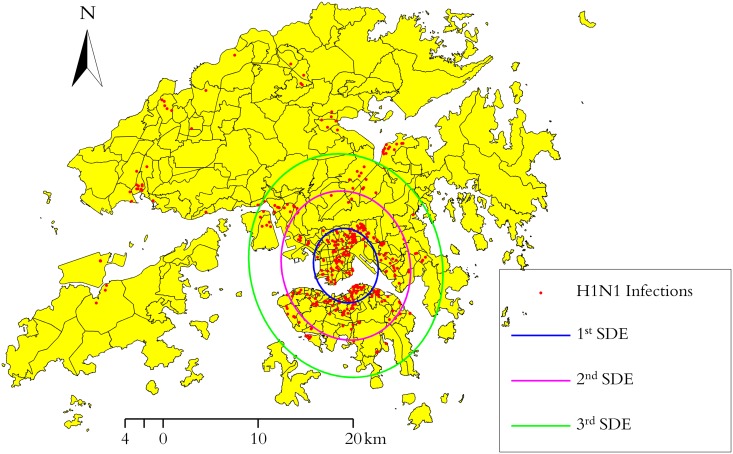
Exploratory analysis by 1–3 multiple SDEs for Hong Kong’s H1N1.

For the epidemic data, totally 410 human swine influenza infected cases are gathered with epidemiological date and address from 1st May to 26th June on a daily basis released by Center of Health Protection (CHP), Hong Kong. Addresses of infected buildings are then geocoded into the WGS84 coordinate for the subsequent mapping. Exploratory analysis by 1–3 multiple SDEs is then conducted in order to keep the focus limited to only those areas with the most occurrences of infected cases (Fig. 7). Although the resulting map output is simple, yet it conveys a strong message about where is the most severe region of H1N1 occurring.

**Fig 8:**
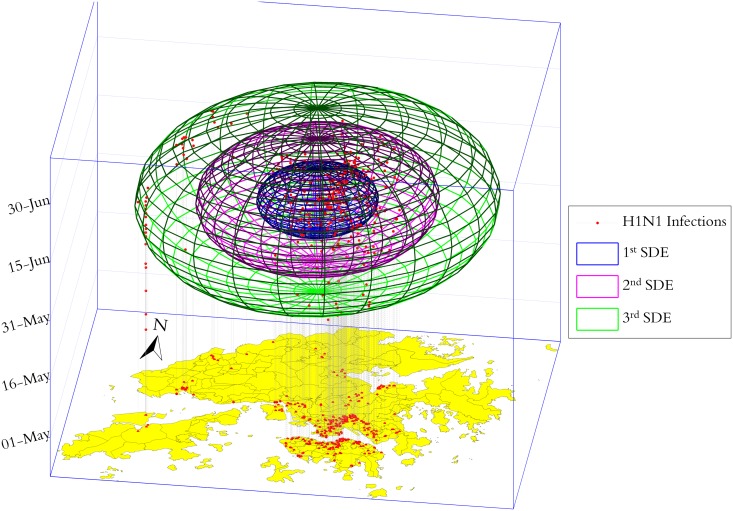
Exploratory analysis by 1–3 multiple SDHEs for Hong Kong’s H1N1.

Further, 1–3 multiple SDHEs (in three-dimensional space) are also employed for highlighting the spatiotemporal concentrations of H1N1 infections (Fig. 8). Apparently, most of the confirmed cases appeared densely during late June in time and converged on both sides of Victoria Harbor, including the Kowloon Peninsula and Hong Kong Island, in space.

## Conclusions

In this paper, confidence analysis of standard deviational ellipse (SDE) and its extension into higher dimensional Euclidean space has been comprehensively explored from origin, formula derivations to algorithm implementation and applications. Firstly, two existing models are summarized and one novel approach is proposed based on the spectral decomposition of sample covariance for calculating the same SDE. After that, the SDE concept is naturally generalized into higher dimensional Euclidean space, named standard deviational hyper-ellipsoid (SDHE). Then, rigorous recursion formulas are derived for calculating the confidence levels of scaled SDHE with arbitrary magnification ratios in any dimensional space. Such formula can be employed for tabulating the confidence levels in relation to the magnification ratio and the space dimensionality more efficiently since the results obtained in low dimensional space can still be repeatedly utilized in subsequent higher dimensional spaces, whereas the traditional approach of calculating the chi-square distribution is mainly relying on the complex computation of gamma density function. Besides, an inexact-newton method based iterative algorithm is also proposed for solving the corresponding magnification ratio of a scaled SDHE when the confidence probability and space dimensionality are pre-specified, thereby making a commutatively computation of either the necessary scaled ratio or the confidence level of SDHE when one of these two parameters is given in any dimensional space. These results provide a more efficient manner to supersede the traditional table lookup of tabulated chi-square distribution.

Finally, synthetic data is employed to generate the 1–3 multiple SDEs and SDHEs. And exploratory analysis by means of SDEs and SDHEs are also conducted for measuring the spread concentrations of Hong Kong’s H1N1 in 2009.

It is worth noting, standard deviational ellipses (or the SDHE) derive under the assumption that observed samples follow the normal distribution. Therefore, SDE tool must be employed with a certain degree of caution when measuring the geographic distribution of concerned features. Particularly, delineation of an area concerned by SDE may not be representative of the hotspot boundaries, but produce ambiguous outcomes when distribution of features is multimodal [12].

Fortunately, the aforementioned normal distribution assumption is no longer indispensable for the confidence ellipses owning to considerable progresses in the last three decades. Nonetheless, these shining ideas emerged during the SDE derivation process still sparkle for prompting innovative advanced models, among which the elliptically contoured distribution [25] attracts wide attention, with its contours of constant density being ellipsoids, that is **(*x***-***μ*)** ^T^ ***C*** ^***-1***^ ***(x***-***μ*) =** *constant*. Amazingly, a scaled SDHE in terms of Eqs. (12)~(15) is actually depicted by this formulation, which also lays core foundation for many of the current popular method, such as the minimum covariance determinant estimator (MCD), multivariate kernel density estimation and support vector machine (SVM) with Gaussian kernel.

## Supporting Information

S1 Table Human cases of swine influenza A (H1N1) gathered with epidemiological date and address from 1st May to 26th June in 2009. (XLSX)
